# An In Vitro Study to Assess the Effect of Cigarette Smoke on Color Stability and Surface Roughness of 3D Printed, Milled, and Traditional Provisional Crown and Bridge Materials

**DOI:** 10.7759/cureus.74505

**Published:** 2024-11-26

**Authors:** Saurabh Jain, Huda A Daak, Nebras E Hamed, Atyaf Fassal Abu Eishah, Abhishek Apratim, Baylasan A Hakami, Shatha Ahmad M Jafari, Renad Hussain M Arjee, Amnah Hadi A Shajiri, Samar Tannous

**Affiliations:** 1 Prosthetic Dental Sciences, College of Dentistry, Jazan University, Jazan, SAU; 2 College of Dentistry, Jazan University, Jazan, SAU; 3 Prosthodontics, Mount Royal Dental, Ontario, CAN; 4 General Dentistry and Comprehensive Care, NYU College of Dentistry, New York University, New York, USA

**Keywords:** ‎3d printing, cad-cam milling, cigarette smoke, color stability, milling, polymethylmethacrylate (pmma), provisional crown and bridge, surface roughness

## Abstract

Objectives

To assess the influence of cigarette smoke (CS) on the color and surface roughness of 3D printed, milled, and traditionally fabricated provisional crown and bridge (PC&B) materials.

Materials and methods

112 disc-shaped samples were made employing four techniques and materials (28 per group) to fabricate PC&B prostheses. Specimens were fabricated using standard protocols, such as 3D printing, milling, conventional bis-acrylic resin, and traditional autopolymerizing polymethyl methacrylate (PMMA) resin. After preliminary color and surface roughness recording, each group specimen was divided randomly into two subgroups (14 each). The artificial saliva acted as the storage media for the control group specimens for 30 days, and test group specimens were subjected to CS in a customized smoking chamber (10 minutes twice daily, for 30 minutes). Final color and surface roughness measurements were made. The change in color (∆E00) and surface roughness (∆Sa) were calculated, and the data was tabulated for analysis.

Statistical analysis

One-way analysis of variance was used to analyze the change in color and surface roughness. Post-hoc Tukey HSD test was used for comparison between groups.

Results

The mean ΔE00 and ∆Sa were higher among groups exposed to CS than those exposed to artificial saliva. The maximum change in color was recorded in the autopolymerizing PMMA, whereas the 3D printed resins recorded the minimal change. The traditional bis-acrylic resin recorded the maximum mean surface roughness change, while the milled resin recorded the least change.

Conclusions

Within the study limitations, it can be inferred that when exposed to CS, 3D printed and milled PC&B materials have superior color stability and displayed less change in surface roughness when equated with traditional bis-acrylic and autopolymerizing PMMA resins.

## Introduction

The provisional restorations play an essential role during a fixed prosthodontic treatment. They shield the pulp, preserve periodontal health, stabilize occlusion, provide aesthetics, and are used as diagnostic aids [1,2]. These restorations should possess the necessary physical and mechanical properties, mainly when intended for use as long-term provisional prostheses, in cases like full-mouth rehabilitation or implant-supported prostheses [2,3].

Commonly used traditional provisional crown and bridge (PC&B) materials include autopolymerizing polymethyl methacrylic (PMMA) resins and bis-acrylic resin [3,4]. They are traditionally used due to low cost and ease of handling. PMMA resin materials have some inherent drawbacks, which include dimensional instability, high polymerization shrinkage, and high residual monomer content, which limits their use in cases that need long-term provisionalization [5,6]. Bis-acrylic resins are biocompatible and have minimal shrinkage, but they are generally used chairside and thus can consume clinical time. Additionally, their outcome will depend on the operator's handling skills [7-9].

With the extensive employment of digital technology in dentistry, milled PMMA-based provisional restorations gained popularity. Due to the fabrication of these discs in commercial labs, these materials have limited free monomer and minimal polymerization shrinkage [5,10]. The limitations of milling include difficulty in milling complex restorations and wastage of material, leading to high fabrication costs [10,11]. 3D printing technology is revolutionary and fabricates prostheses with additive technology. There is minimal wastage of materials, and with the easy availability of printers, the prosthesis can be fabricated at a low cost [12-14]. Studies have shown that these materials have good properties and can be used as PC&B materials [13-15].

Deleterious effects of cigarette smoking (CS) are not limited to the systematic impact; they have also been shown to affect dental restorations within the mouth [16-23]. Published literature has evaluated the impact of CS on various dental restorative materials used in the mouth. Ayaz et al. and Patil et al. assessed the influence of CS on denture teeth, whereas Alandia-Roman et al. and Wasilewski et al. researched dental composite and reported changes in color and surface roughness after exposure to CS [16-19]. Similar results were reported by other studies where the test material was CAD/CAM restorative material, denture soft liners, and denture base materials [20-23]. For any restoration to succeed, it should be aesthetically pleasing and have minimal color change. A color change may be disappointing for the patient, which may doubt the material quality and the prosthesis's serviceability [22]. Similarly, a smooth prosthesis surface is deemed necessary to prevent plaque accumulation. If the surface of the prosthesis becomes rough, it will attract plaque and may cause inflammation.

The current literature lacks published research assessing the impact of CS on tested provisional materials. Thus, the current research aims to assess the effect of CS on the color and surface roughness of 3D printed, milled, and traditional PC&B materials. The null hypotheses are 1) CS has no effect on the color and surface roughness of tested PC&B materials and 2) all four PC&B materials have similar color and surface roughness changes, if any.

## Materials and methods

The current research evaluated the consequence of exposing four PC&B materials to CS. The parameters assessed were alterations in color and surface roughness. The research protocol was approved by the Scientific Research Committee at the College of Dentistry, Jazan University (Reference No. CODJU-2305F). Four techniques and materials for fabricating PC&B were used to fabricate the specimens, which were later exposed to CS under controlled conditions, simulating the oral cavity.

**Figure 1:**
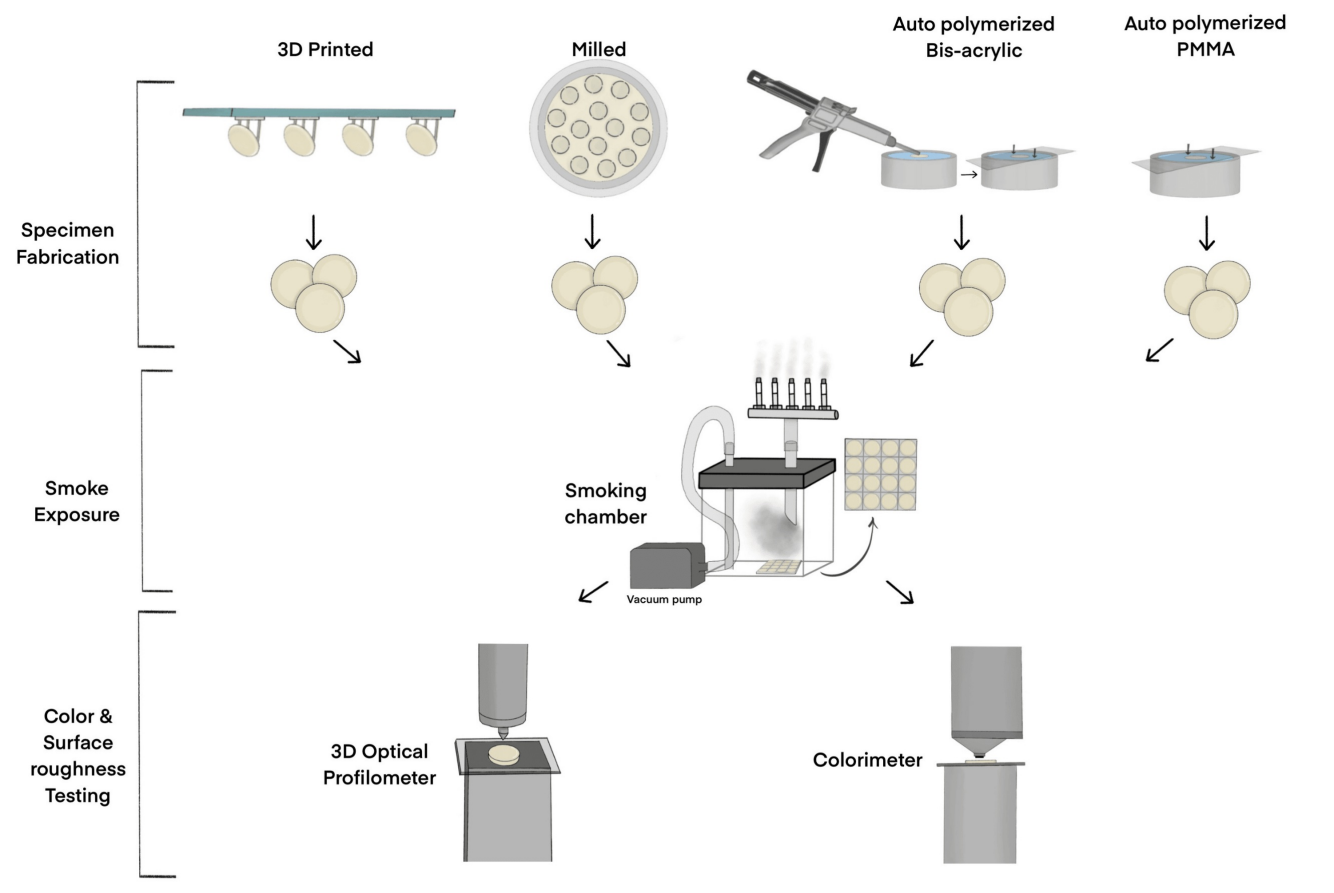
Schematic representation of steps involved in the study.

G-power software (version 3.1.9.7, 2020; Heinrich Heine University, Germany) was employed for calculating the sample size. Data from a previously published article was used for calculating the effect size [23]. With power at 90%, alpha value at 5%, and effect size (f) at 0.35, the sample of 14 per group was found to be appropriate. One hundred and twelve specimens (disc-shaped, dimension: 2 mm height x 10 mm diameter) were fabricated using four techniques used for PC&B fabrication (milling, 3D printing, and traditional methods (bis-acrylic, and autopolymerizing resin)). Independent variables will be PC&B material and CS, whereas dependent variables will be alterations in color and surface roughness. Figure 1 is the schematic representation of the steps followed in the study.

Specimen fabrication

For fabricating 28 milled specimens, CAD software (Microsoft 3D builder, USA) was employed for designing the specimen virtually. This STL file was used for dry milling the prepolymerized block of PMMA-based crown and bridge resin (Telio CAD, Ivoclar Vivadent) using Opera Pro-Expert-5 (Euromax Monaco, Monaco). This STL file was subsequently utilized again to produce 3D printed samples. The manufacturer's recommendations (layer thickness: 50 µm; printing orientation: 45°) were followed while using the 3D printer (NextDentTM 5100, NextDent B.V.). Twenty-eight specimens were printed using the resin Next Dent C&B MFH (Vertex-Dental BV), which were later cleaned and light-cured. For fabricating the specimens using traditional techniques, a putty index was fabricated and used as a mold in which nonpolymerized bis-acrylic resin (Protemp 4, 3M ESPE) and autopolymerizing PMMA resin (DPI self-cure tooth molding acrylic) were kept. A glass slab was used to cover the top of the mold, which will aid in getting a smooth surface [24]. So, 28 bis-acrylic resin specimens and 28 PMMA resin specimens were fabricated.

Abrasive carbide discs (Buehler Ltd., USA) were used for finishing, and universal polishing paste was used for polishing the specimens. One researcher did all the procedures. For each group, five extra specimens were prepared to replace the poor-quality specimens after evaluation using dental loupes.

Preliminary measurements

A trained operator recorded all the specimens' preliminary color and surface roughness measurements under standardized conditions. For color measurement, a portable colorimeter (CS-10, Hangzhou Quality Lab Scientific Co. Ltd., China) was used. The instrument was standardized as per the company’s specifications, and the specimen was positioned on a white background (to ensure accurate measurement of color parameters by effectively minimizing possible absorption effects) [24,25]. Three color readings, one from the middle and two haphazardly from other parts, were recorded and averaged. For surface roughness measurements, a 3D optical non-contact surface profiler (UMT 1, Campbell, USA) was used, and the average of three readings was calculated.

Exposure to cigarette smoke

**Figure 2:**
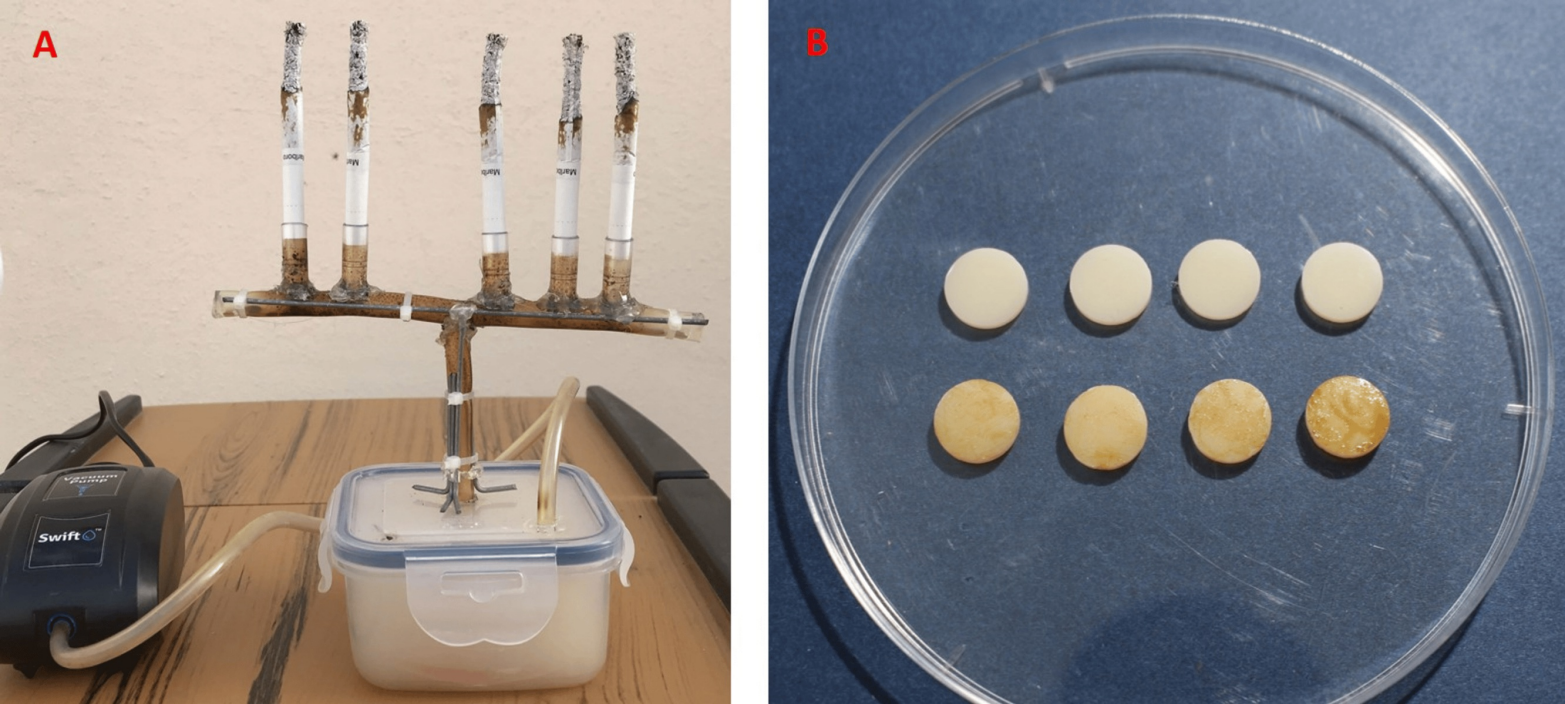
A) Smoking chamber used in the study. B) Specimens after exposure (control group and test group specimens).

A simple randomization technique was employed using a computer-generated random number to divide the specimens into a control and an experiment group (n=14 per group) [26]. The artificial saliva acted as the storage media for the control group specimens for 30 days (the solution was replaced every day). For the experiment group, a customized smoking chamber was used, and the specimens were placed and exposed to CS, simulating 30 days of exposure. A custom-made smoking chamber was fabricated and designed based on previously published studies (Figure 1, 2A) [22,27]. A five-port cigarette holder was fixed to one end, whereas a vacuum pump was attached to the other end, which helps in smoking the cigarettes by generating a negative pressure (approximately 2660 Pa) [22]. The specimens were placed in the chamber, cigarettes (Marlboro Gold, Philip Morris, Switzerland) were smoked up to 10 mm from the filter, and the chamber was left filled with smoke for 10 minutes [19,22]. The specimens were rinsed and placed in artificial saliva. This process was performed twice daily for 30 days (Figure 1; Figure 2B).

Final measurements

The protocol used for recording preliminary color and surface roughness measurements was repeated to record the final measurements after exposure of specimens to CS. The operator was blinded to avoid operator bias. The CIE ΔE2000 formula was employed to compute the difference in the color [28].

∆E00 = [ (∆L* /KL × SL)2 + (∆C* /KC × SC)2 + (∆H* /KH × SH)2 + RT × (∆C* /KC × SC) × (∆H* /KH × SH)]1/2

Here ΔL* denotes the difference in brightness, ΔC* denotes the difference in saturation, and ΔH* denotes the difference in hue between the preliminary and final color readings. Corrections were performed using weighing coefficients (SL, SC, and SH) and parametric coefficients (kL, kC, and kH) as constants [28].

The formula for calculating the difference in surface roughness was ∆Sa = Sa1 − Sa0. Here Sa0 and Sa1 denote the preliminary and the final surface roughness, respectively, and ∆Sa represents the difference in µm.

Data analysis

The collected data was entered and analyzed using SPSS software (version 26, IBM Corp., USA). Almost all the data were normally distributed (Shapiro-Wilk test (p-value ≥0.05)). However, the mean and standard division were calculated. The independent t-test was used to test the significant difference between cigarette smoking and artificial saliva (control) groups. Moreover, one-way ANOVA was used to test the significant difference between four PC&B materials. Moreover, a post-hoc Tukey HSD test was used for comparison between every two groups. A p-value <0.05 was considered as a cut-off point for statistical significance.

## Results

Four groups of PC&B materials, each containing 14 samples, were exposed to both CS and artificial saliva (control group). These groups were categorized and stratified into eight subgroups based on material type and exposure conditions. Changes in color stability and surface roughness were detected and recorded.

Change in color

**Figure 3:**
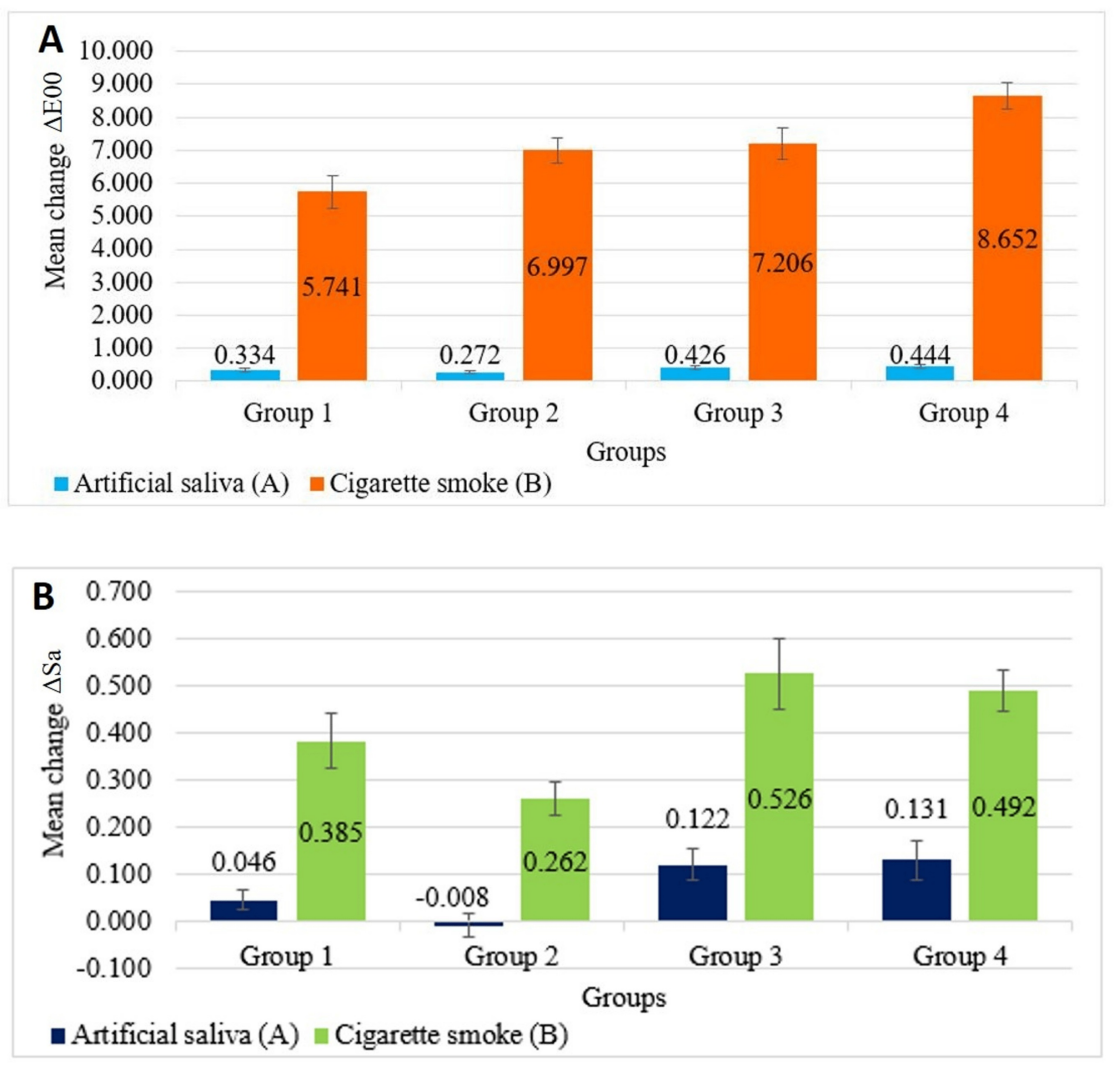
A) The mean change in color (ΔE00) after exposure to CS. B) The mean change in surface roughness (ΔSa (µm)) after exposure to CS. CS, cigarette smoke

Figure 3A shows that the mean color change (ΔE00) among groups exposed to CS (B) was higher than that among groups exposed to artificial saliva (A). Regarding materials exposed to CS, the highest mean color change was recorded in Group 4B (8.652 ±0.383), while the least mean change was recorded in Group 1B (5.741 ± 0.503). However, among materials exposed to artificial saliva, the highest mean color change was recorded in Group 4A (0.444 ± 0.053), while the least mean change was recorded in Group 2A (0.272 ± 0.040).

The change in color for all four groups of PC&B materials exposed to CS was significantly higher, compared to those exposed to artificial saliva (p-values <0.0001). Group 4 showed the highest color change (8.208), indicating that it is most susceptible to color change from CS. However, Group 1 had the least color change (5.407), indicating that it is least susceptible to color change from CS (Table 1). Moreover, the results showed highly significant differences in color stability of the four material groups between and within the two groups (artificial saliva and CS), (p-values <0.0001).

**Table 1:** Comparison of the mean change in color (ΔE00) between and within artificial saliva and CS groups. ^a^Independent t-test ^b^One way ANOVA CS, cigarette smoke; PC&B, provisional crown and bridge; df, difference

PC&B material	Artificial saliva (Control group), mean (±SD)	CS, mean (±SD)	df	t-value	P-value^a^
Group 1 (3D Printed)	0.334 (±0.046)	5.741 (±0.503)	5.407	40.027	<0.0001
Group 2 (Milled)	0.272 (±0.040)	6.997 (±0.368)	6.725	67.946	<0.0001
Group 3 (Conventional Bis-acrylic)	0.426 (±0.056)	7.206 (±0.469)	6.780	53.731	<0.0001
Group 4 (Conventional PMMA)	0.444 (±0.053)	8.652 (±0.383)	8.208	79.512	<0.0001
Mean square	0.091	19.913			
F	37.954	105.466			
P-value^ b^	<0.0001	<0.0001			

Table 2 shows a post hoc test comparing two different groups of materials. There were statistically significant differences in color change between Group 1A vs Groups 2B, 3B, and 4B (p-values <0.0001), Group 1B vs Groups 2A, 3A, and 4A (p-values <0.0001), Group 2A vs Groups 3B and 4B (p-values <0.0001), Group 2B vs Groups 3A and 4A (p-values <0.0001), Group 3A vs Group 4B (p-value <0.0001), and Group 3B vs Group 4A (p-value <0.0001). However, the largest difference in color change was found between Group 2A vs Group 4B (8.380), followed by Group 1A vs Group 4B (8.318), and Group 3A vs Group 4B (8.226).

**Table 2:** Post hoc test for comparing the change in color (ΔE00) and surface roughness (ΔSa) between each pair of material groups.

Change in color (ΔE00)	Difference	Std. error	P-value
Group 1A vs Group 2B	6.664	0.116864	<0.0001
Group 1A vs Group 3B	6.872	0.116864	<0.0001
Group 1A vs Group 4B	8.318	0.116864	<0.0001
Group 1B vs Group 2A	5.469	0.116864	<0.0001
Group 1B vs Group 3A	5.315	0.116864	<0.0001
Group 1B vs Group 4A	5.297	0.116864	<0.0001
Group 2A vs Group 3B	6.934	0.116864	<0.0001
Group 2A vs Group 4B	8.380	0.116864	<0.0001
Group 2B vs Group 3A	6.572	0.116864	<0.0001
Group 2B vs Group 4A	6.554	0.116864	<0.0001
Group 3A vs Group 4B	8.226	0.116864	<0.0001
Group 3B vs Group 4A	6.762	0.116864	<0.0001
Change in surface roughness (ΔSa)	Difference	Std. error	P-value
Group 1A vs Group 2B	0.216	0.016867	<0.0001
Group 1A vs Group 3B	0.480	0.016867	<0.0001
Group 1A vs Group 4B	0.446	0.016867	<0.0001
Group 1B vs Group 2A	0.393	0.016867	<0.0001
Group 1B vs Group 3A	0.262	0.016867	<0.0001
Group 1B vs Group 4A	0.254	0.016867	<0.0001
Group 2A vs Group 3B	0.534	0.016867	<0.0001
Group 2A vs Group 4B	0.500	0.016867	<0.0001
Group 2B vs Group 3A	0.140	0.016867	<0.0001
Group 2B vs Group 4A	0.132	0.016867	<0.0001
Group 3A vs Group 4B	0.370	0.016867	<0.0001
Group 3B vs Group 4A	0.396	0.016867	<0.0001

Change in surface roughness (ΔSa)

Figure 3B shows that the change in surface roughness (ΔSa) for specimens exposed to CS (B) was higher than for the specimens exposed to artificial saliva (A).

**Figure 4:**
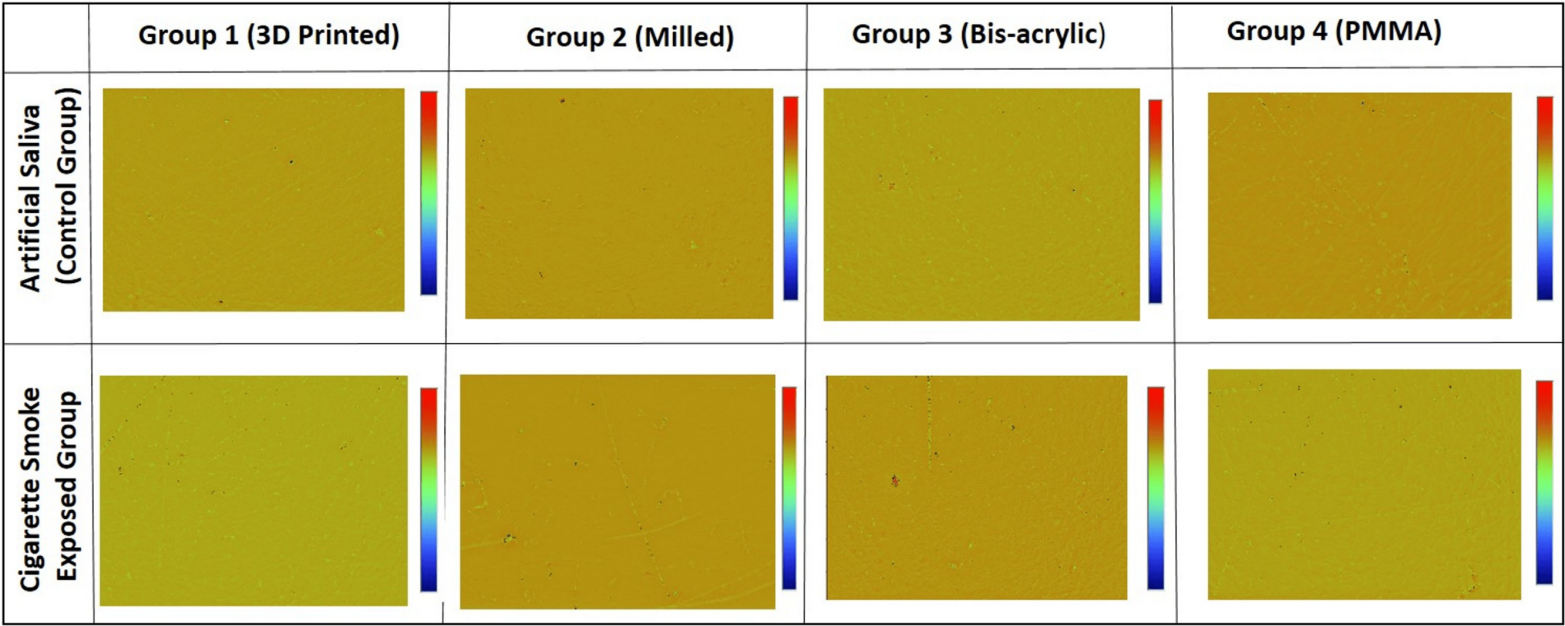
3D images of graphical output of the surface of different PC&B materials used in the study. PC&B, provisional crown and bridge

Concerning materials exposed to CS, the maximum change in surface roughness was detected in Group 3B (0.526 ± 0.076), while the minimum change was detected in Group 2B (0.262 ± 0.036). However, among materials exposed to artificial saliva, the maximum change in surface roughness was detected in Group 4A (0.131 ± 0.041), while the least mean change was observed in Group 2A (-0.008 ± 0.024) (Figure 4).

Table 3 shows the change in surface roughness of all four groups of PC&B materials exposed to CS was significantly higher, compared to those materials exposed to artificial saliva (p-values <0.0001). Group 3 showed the largest difference in surface roughness change (0.404), indicating the most obvious impact of CS on its surface roughness. However, Group 2 showed the smallest difference in surface roughness change (0.270), indicating the least obvious impact of CS on its surface roughness. Additionally, there were statistically significant differences in surface roughness of the four material groups between and within the artificial saliva and CS groups (p-values <0.0001).

**Table 3:** Comparison of the mean change in surface roughness (ΔSa) between and within artificial saliva and CS groups. ^a^Independent t-test ^b^One way ANOVA CS, cigarette smoke; PC&B, provisional crown and bridge; df, difference

PC&B material	Artificial saliva (Control group), mean (±SD)	CS mean (±SD)	df	t-value	P-value^ a^
Group 1 (3D printed)	0.046 (±0.020)	0.385 (±0.058)	0.339	20.602	<0.0001
Group 2 (milled)	-0.008 (±0.024)	0.262 (±0.036)	0.270	23.141	<0.0001
Group 3 (conventional Bis-acrylic)	0.122 (±0.032)	0.526 (±0.076)	0.404	18.467	<0.0001
Group 4 (conventional PMMA)	0.131 (±0.041)	0.492 (±0.043)	0.361	22.735	<0.0001
Mean square	0.061	0.199			
F	66.703	64.645			
P-value ^b^	<0.0001	<0.0001			

Table 2 shows a post hoc test comparing two different groups of materials. There were statistically significant differences in surface roughness change between Group 1A vs Groups 2B, 3B, and 4B (p-values <0.0001), Group 1B vs Groups 2A, 3A, and 4A (p-values <0.0001), Group 2A vs Groups 3B and 4B (p-values <0.0001), Group 2B vs Groups 3A and 4A (p-values <0.0001), Group 3A vs Group 4B (p-value <0.0001), and Group 3B vs Group 4A (p-value <0.0001). However, the largest difference in surface roughness change was found between Group 2A vs Group 3B (0.534), followed by Group 2A vs Group 4B (0.500), and Group 1A vs Group 3B (0.380).

## Discussion

The current study assessed the change in color and surface roughness of four PC&B materials after exposure to CS. The results of the change in color revealed that the maximum mean change was recorded in the autopolymerizing PMMA resin group, whereas the minimum mean change was recorded in the 3D printed group. For the surface roughness, the maximum mean change was seen in the bis-acrylic group, while the least mean change was detected in the milled group. Thus, both the null hypotheses that were tested were rejected. However, the magnitude of change in color and surface roughness varied among the groups.

Among the various forms of tobacco intake, cigarette smoking is one of the most typical forms. Multiple studies have reported the effect of CS on various dental materials but its effect on milled and 3D printed PC&B materials has not yet been documented [16-23].

The present study used a custom-made smoking chamber. A vacuum pump was used to create a standardized negative pressure, and five cigarettes were smoked at the same time to create smoke in the chamber [19,22]. Exposure time to CS and other conditions was kept in accordance with previous published studies [19,22]. A few specimens representing each group were kept simultaneously to confirm that all specimens were exposed to the same testing situations.

Color and surface roughness are two critical parameters for any dental restoration. A rough surface of the PC&B can lead to faster biofilm formation, leading to gingival inflammation [4-6]. Similarly, a PC&B prosthesis should be color-stable, especially if used as a long-term provisional prosthesis. Perceivable change in color can be unacceptable for the patient, who may relate it to the quality of the dental treatment and materials used [22].

In the current research, a colorimeter was utilized to quantify the change in color, as this is a more acceptable technique for objectively measuring color [29,30]. The current study employed the CIE2000 formula for calculating the change in color (ΔE2000). Studies have reported that the CIELAB2000 formula is superior to the conventional CIELAB formula in evaluating a change in color by humans [28]. In the current study, the perceptible threshold of color difference was kept at ∆E00<1.2 while the acceptability threshold of color difference was kept at delta ∆E00<2.7 [25,31].

In the present study, a higher change in color was reported for specimens exposed to CS compared to the control group. For the control group, all the values were far below the clinically perceptible threshold values, whereas for all the CS group specimens, changes in color values were above the clinically acceptable threshold values. This higher change in color can be reported due to the deposition of tannins and other minerals present in the CS on the surface of tested specimens [16-18]. The results of our study cannot be compared directly to previously published studies. The results were in accordance with studies by Ayaz et al., Sayed et al., and Schelkopf et al., who reported similar results, although the tested specimens were different (denture teeth, soft denture liners, and CAD/CAM materials, respectively) [16,21,22].

When the color change was compared between the four tested materials, the highest change was reported in traditional PMMA resin, followed by bis-acrylic and milled resin. 3D printed specimens reported the least color change. The current study's findings cannot be directly compared to previously published studies. Our study partially agrees with Sayed et al., who evaluated the impact of smokeless tobacco on a similar group of materials and reported that the maximum change in color was reported in PMMA resin PC&B specimens (∆E = 9.343 ± 0.489), whereas the least change was reported in milled PC&B specimens (∆E = 4.978 ± 0.227) [22]. A lower change in the color of milled specimens could be due to the difference in the manufacturing process. The CAD/CAM resin blocks are commercially manufactured under high heat and pressure and thus are highly compact [5,15]. Whereas autopolymerized PMMA resin has high porosities and higher free monomer quantity, thus leading to higher discoloration when exposed to CS [16,32].

In this research, higher changes in color were reported by milled resin specimens than by 3D printed ones. These are not in agreement with the studies of Shin et al., Tasın et al., and Sayed et al., who reported contrasting results [24,33,34]. This was attributed to more gaps in the layers of 3D printed resins compared to milled resins [35]. The current research was in partial agreement with Tasın et al., who exposed PC&B materials to staining solutions and reported lower alteration in color for 3D printed resins in comparison to traditional PMMA resins but higher than milled resins [34]. This could be attributed to the incorporation of extra surface defects during the milling process, compared to 3D printed resins, which are polymerized, thus improving surface smoothness [36]. The difference could also be due to the fact that in all the previous studies, the specimens were immersed in liquid solutions (which can get absorbed), whereas in the current study, they were exposed to CS. In the present study, bis-acrylic resins displayed lower changes in color than autopolymerizing PMMA resins. This was in accordance with previous studies by Sham et al. and Bitencourt et al., who reported similar changes but were contrary to results reported by Rayyan et al. and Elagra et al. [3,5,37,38]. This was attributed to the molar polar structure of bis-acrylic resins and the use of an auto-mixing system, leading to lower surface defects [3,5,37,38].

The non-contact profilometer used in this study records 3D surface roughness (Sa) without physically touching the surface of the material, thus avoiding scratch formation on the tested surface [39]. One researcher performed all the specimen polishing in a uniform manner to ensure that any alteration in surface roughness was attributed to the specimens' exposure to the CS [39].

After exposure to CS, there was a significant increase in surface roughness of all the tested specimens but to a varied extent. The control group specimens reported minimal change in surface roughness. This could again be related to the accumulation of tannins and minerals on the surface of the specimens which, in addition to the heat of the CS, may change the surface topography of the exposed materials [16-18]. Previous literature assessing the effect of CS on different materials reported an increase in surface roughness of the tested materials after exposure to CS [16,18,23]. In the present study, the highest change was reported in bis-acrylic (0.526 (±0.076)) followed by PMMA (0.492 (±0.043)), 3D printed (0.385 (±0.058)), whereas milled displayed the lowest change (0.262 (±0.036)). In all the materials, the change in roughness was beyond the acceptable threshold of 0.2 μm [40]. Due to a lack of similar previous published research work, direct comparison is not possible. These findings were consistent with the study by Sayed et al., who, when exposed to the PC&B resins to smokeless tobacco, reported the highest changes in bis-acrylic resins and the lowest changes in milled resins [24]. These were inconsistent with the study by Tasin et al., who documented the lowest change in surface roughness for 3D printed resins, followed by traditional bis-acrylic resins, milled resin, and the highest change in traditional PMMA resins, after exposing them to staining solutions [34]. Reduced porosity and minimal free monomer in milled resins could be attributed to a lower change in the surface roughness of these materials [5,15].

The present study has a few limitations, including its in vitro nature, which makes it difficult to imitate the same clinical conditions to which these PC&B resin materials are exposed. The effect of other food particles, brushing, and masticatory forces cannot be replicated. Additionally, the study is limited to only one shade of one commercial brand of each type of material. Furthermore, in vivo studies with more brands of materials should be conducted in the future.

## Conclusions

Within the study limitations, it can be inferred that 3D printed and milled PC&B resins have better color stability and less change in surface roughness compared to traditional bis-acrylic and autopolymerizing PMMA resins when exposed to CS. This study can help dentists in choosing the PC&B materials for smoking patients who need long-term provisional restorations. 
